# The rescue intervention strategy for asthma patients under severe air pollution: a protocol for a single-centre prospective randomized controlled trial

**DOI:** 10.1186/s13063-020-04830-0

**Published:** 2020-11-04

**Authors:** Xiaoyu Yang, Junjun Huang, Yan Hu, Cuiyan Guo, Xi Wang, Zhao Yang, Tianyu Zhou, Guangfa Wang

**Affiliations:** 1 Department of Respiratory and Critical Care Medicine, Peking University First Hospital, Beijing, 100034 China; 2 Department of Scientific Research, Peking University First Hospital, Beijing, 100034 China

**Keywords:** Asthma, Exacerbation, Air pollution, Budesonide/formoterol, Rescue intervention strategy

## Abstract

**Background:**

Asthma is a common chronic airway inflammatory disease. Exacerbations of asthma not only accelerate the progression of the disease but also increase the incidence of hospitalization and death. Studies have shown that air pollution is a high-risk factor for asthma exacerbations. However, few treatment strategies have been recommended to reduce the risk of severe air pollution-related asthma exacerbations.

**Methods/design:**

This is a single-centre, prospective, randomized and standard treatment parallel control clinical trial. Seventy-two asthma patients in the nonexacerbation stage according to GINA guidelines 2017 will be recruited and randomized into the rescue intervention strategy (RIS) group and control group. Original treatments for the participants will include no use of inhaled medicine, the use of short-acting β-agonists (SABA) on demand or the use of budesonide/formoterol (160 μg/4.5 μg/dose, 1–2 dose/time, b.i.d.). The rescue intervention strategy for the RIS group will be budesonide/formoterol plus the original treatment until the severe pollution ends (air quality index, AQI < 200). The control group will maintain the original treatment. The follow-up observation period will last 1 year. The primary outcome is the frequency of asthma exacerbations per year. Secondary outcomes include the mean number of unplanned outpatient visits, emergency visits, hospitalizations, medical costs and mortality caused by asthma exacerbations per patient per year.

**Discussion:**

The results of this trial will provide a novel strategy to guide clinical practice in decreasing the risk of asthma exacerbations under severe air pollution.

**Trial registration:**

ChiCTR ChiCTR1900026757. Registered on 20 October 2019—retrospectively registered

## Introduction

Recently, increasing attention has been drawn to air pollution and its serious consequences, especially in China and other developing countries. The evidence has demonstrated that air pollution could cause critical public health problems. A retrospective study of 80,515 deaths in Beijing during 2004–2008 found that the reduction in life expectancy was associated with increased air pollution. More specifically, an interquartile range increase in particulate matter with aerodynamic diameter < 2.5 μm (PM_2.5_), PM_10_, SO_2_ and NO_2_ was associated with 15.8, 15.8, 16.2 and 15.1 years of life lost, respectively [1].

Asthma is a common chronic airway inflammatory disease, with more than 45 million adults suffering from asthma in China [2]. Exacerbations of asthma not only accelerate the progression of the disease but also increase the incidence of hospitalization and death. It has already been proven that air pollution can cause asthma exacerbations [3]. Unfortunately, few treatment strategies have been recommended to reduce severe air pollution-related asthma exacerbations. Inhaled corticosteroids (ICS)/long-acting β-agonists (LABA) with single maintenance and relief therapy (SMART) are well known for significantly reducing asthma exacerbations [4]. However, only when patients have symptoms will SMART be applied, meaning that the airways have already been damaged by atmospheric pollutants and the subsequent inflammatory response. Some treatments with ICS/LABA (such as budesonide/formoterol) might stop these inflammatory responses with rapid action [5].

Therefore, we hypothesize that the rescue intervention strategy of budesonide/formoterol plus original treatments under severe pollution may reduce the risk of asthma exacerbations caused by air pollution before patients have symptoms. We will undergo a 1.5-year single-centre prospective randomized controlled clinical trial to compare the frequency of asthma exacerbations per year and asthma exacerbation-related visits, hospitalizations, mortality, medical costs etc. between the rescue intervention strategy (RIS) group and the control group.

## Methods

### Study design and setting

**Fig. 1:**
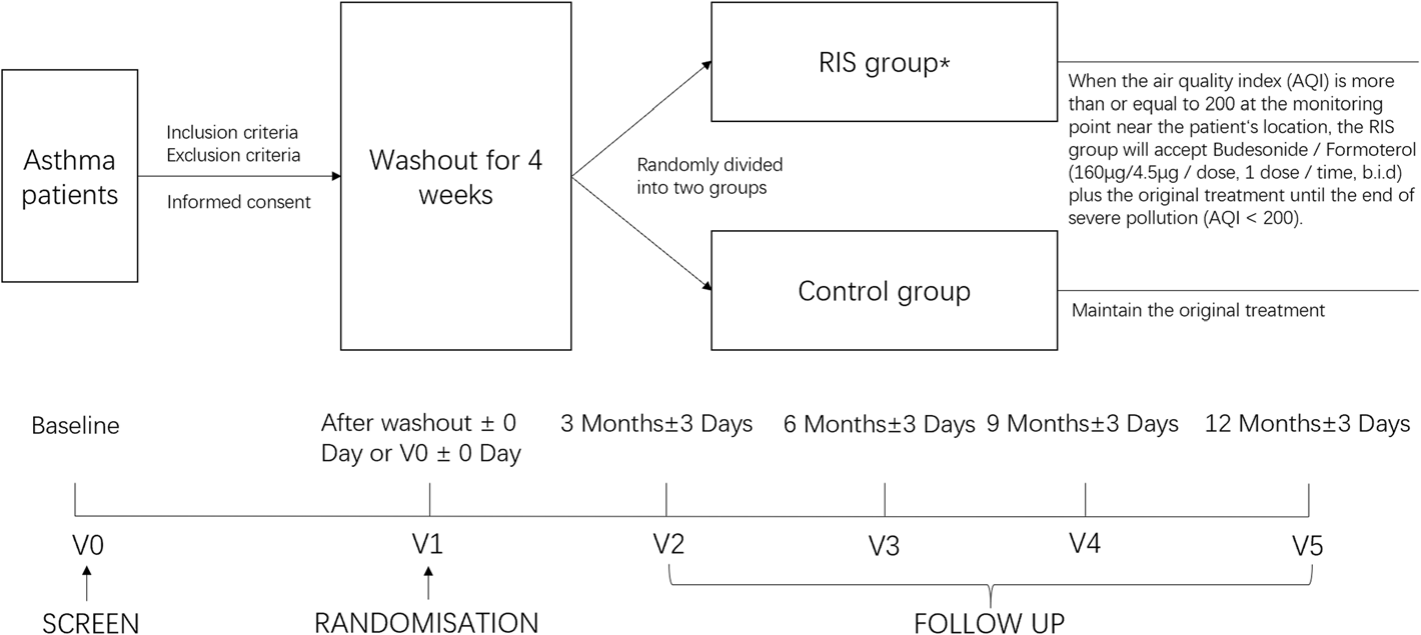
The study design flowchart (*RIS group, rescue intervention strategy group)

This study is a single-centre, prospective, randomized and standard treatment parallel control clinical trial (see Fig. 1). We followed the standardized programme intervention: Standard Protocol Item Recommendations for Interventional Trials (SPIRIT) 2013. We followed similar methods of Zhou et al. 2019 [6], especially in the ‘Intervention’ and ‘Outcomes’ sections. Patients who meet the inclusion criteria (details are shown in the ‘Inclusion criteria’ section) and do not meet the exclusion criteria (details are shown in the ‘Exclusion criteria’ section) will be recruited from Peking University First Hospital. Advertising strategies such as posters, social media and popular community websites will be used to increase recruitment. When screening, the purposes, procedures, potential benefits and risks of the study will be explained carefully by the investigators. After explaining the trial, the investigators will answer any questions that the participants may have about the study. Participants will then make the final decision on whether to participate and sign the informed consent form. Then, written informed consent will be obtained, and each participant visit will be overseen by a trained clinician.

At the baseline visit (V0), basic data will be collected, including sex, age, education, income, type of medical insurance, workplace/home addresses, the air pollution monitoring station for the study (which is defined as the nearest air pollution monitoring station from the workplace for employees or from home for nonemployees), medical/surgical history, suspected allergen contact history (such as pets), therapeutic scheme for asthma and any exacerbations experienced within the past 3 months. Physical examinations will also be performed (height, weight, body mass index (BMI), heart rate and blood pressure), along with the Chinese version of asthma assessment scales, which have shown good validity [7] (the Mini Asthma Quality of Life Questionnaire 7 (mini-AQLQ 7), the Numerical Control Questionnaire (ACQ) and Asthma Control Test (ACT)), lung function testing (bronchodilator reversibility test) and fraction of exhaled nitric oxide (FeNO) measurement, which will be interpreted with the use of cut points.

Several interventions, such as no use of inhaled medication, the use of SABA on demand or the use of budesonide/formoterol (160 μg/4.5 μg/dose, 1–2 dose/time, b.i.d.), are acceptable as the original treatment in our study. Participants receiving other treatments must enter a washout period (see Table 1). Thereafter, they will be randomly divided into two groups: the RIS group and the control group. Exacerbation situations within the past 3 months, physical examinations, asthma assessment scales, lung function tests (bronchodilator reversibility tests) and FeNO measurements will be repeated on the randomization day (V1).

**Table 1:** The therapeutic replacement scheme for the washout period

Therapeutic scheme before the washout period	Therapeutic replacement scheme	Washout period
No use of inhaled medication	Maintain the original treatment	Go directly into randomization without washout
SABA on demand		
Budesonide/formoterol (160 μg/4.5 μg/dose, 1–2 doses/time, b.i.d.)		
Regular inhalation of SABA and/or LABA and/or ICS and/or anticholinergics and/or other types/doses of inhaled drugs	Budesonide/formoterol (160 μg/4.5 μg/dose, 1–2 doses/time, b.i.d.)	For 4 weeks

*SABA* short-acting β-agonists, *LABA* long-acting β-agonists, *ICS* inhaled corticosteroids

When the air quality index (AQI) reported by the air pollution monitoring station for the study is no less than 200, participants in the RIS group will receive budesonide/formoterol (160 μg/4.5 μg/dose, 1 dose/time, b.i.d.) plus original treatments until the end of severe pollution (AQI < 200). At the same time, participants in the control group will continue to receive the original treatment.

Participants will visit Peking University First Hospital every 3 months and will be followed up for 1 year (V2–V5). At each visit, exacerbations within the past 3 months, physical examinations, asthma assessment scales and lung function tests (bronchodilator reversibility tests) will be repeated. FeNO measurements will be repeated only at the final visit (V5).

### Inclusion criteria

The inclusion criteria were as follows: (1) age between 18 and 80 years old (male or female), (2) asthma patients at the nonexacerbation stage (according to GINA guidelines 2017), (3) smoking cessation for more than or equal to 6 months or no smoking history, (4) no restrictions in performing daily activities, (5) a resident of Beijing (employees should make sure that there are air pollution monitoring stations within 5 km of their workplace, and nonemployees should make sure that there are air pollution monitoring stations within 5 km of their home), (6) a smartphone available at their disposal, (7) willing to provide written informed consent and (8) willing to follow the research programme.

### Exclusion criteria

The exclusion criteria were as follows: (1) diagnosis of another chronic respiratory diseases, such as chronic obstructive pulmonary disease, lung cancer, tuberculosis, bronchiectasis and diffuse lung disease (interstitial pneumonia, occupational lung disease, sarcoidosis etc.); (2) a history of lobectomy, lung transplantation or pleural disease; (3) severe underlying disease (including severe psychiatric disorders, dysgnosia, nervous system disease, other malignant tumour, chronic liver disease, heart failure, autoimmune disease and chronic kidney disease); (4) life expectancy of less than 3 years; (5) no participation in outdoor activities; (6) expecting to move out of Beijing within 2 years; (7) planning to decorate home or workplace during the research period; (8) alcohol or substance abuse; (9) allergy history or other contraindication against the medicine used in this trial; (10) participating in other clinical trials; (11) poor compliance; (12) unwilling to provide written informed consent; (13) diagnosis of osteoporosis or diabetes due to the risk of adverse effects related to the use of budesonide/formoterol; and (14) cigarette smoking more than or equal to 10 pack-years.

To estimate compliance before the start of the study, investigators will describe the process of our study in detail and emphasize the long-term follow-up when screening. Patients will answer three questions with ‘yes’ or ‘no’. The questions are ‘Over the past two weeks, were there any days when you forgot to take your asthma medicine?’, ‘Taking medication every day is a real inconvenience for some people. Do you ever feel hassled about sticking to your asthma treatment plan?’ and ‘Do you have difficulty remembering to take all your asthma medication?’. If the answers are all ‘yes’, the patient’s compliance will be assessed as poor.

### Randomization and grouping

Block randomization will be used to generate random codes. The random codes will be designed in a 1:1 ratio (RIS group or control group) using the SAS 9.2 software package (SAS Institute, Cary, NC). A researcher who is not participating in this study will generate random codes and make random grouping envelopes based on the generated results. The envelopes will be sealed and handed to the researcher responsible for grouping. After screening, participants will be identified by subject numbers according to the sequence in which they enter this trial. When grouping, the envelope with the corresponding number for the subject number of the participant will be opened. The participant will be assigned to the RIS group or the control group according to the randomization result in the envelope. The randomization result will be just told to the participant and researchers responsible for grouping and intervention, then it will be resealed in the same envelope until the end of the study.

### Intervention

After randomization and grouping, all participants will be asked to add WeChat (a popular social app provided by Tencent Company, China) friends with the intervention clinician. The communications between participants and the intervention clinician will be mainly through the WeChat app. The data in this app will be saved and backed up.

The real-time AQI will be collected from the Beijing Air Pollution app (provided by the Beijing Municipal Environmental Monitoring Center, China). The intervention clinician will send the real-time AQI from the air pollution monitoring station for the study to each participant via WeChat between 9 AM and 10 AM every day. When the AQI is no less than 200, the intervention clinician will ask the participants in the RIS group to take budesonide/formoterol (160 μg/4.5 μg/dose, 1 dose/time, b.i.d.) plus the original treatment until severe pollution ends (AQI < 200), as indicated by WeChat. These participants will reply to the intervention message as a confirmation via WeChat. At the same time, the control group will be asked to focus on protective strategies (avoid outdoor activities, for example) and maintain their original treatments. The follow-up observation period will last 1 year. Daily AQI and whether the intervention is successfully accomplished during every intervention period will be recorded by WeChat, and investigators will have a backup copy of these data.

### Follow-up and data collection

When participants need to visit the hospital to receive medicine for asthma or due to respiratory symptoms, they will be asked to visit with the intervention clinician. Then, the intervention clinician will assign a clinician from our study who is working at Peking University First Hospital that day to provide medical services to the participants. Medical records will be completed and saved in the Medical Record System of Peking University First Hospital. The completed medical records will be printed immediately to preserve the data. If the participants visit another hospital for an emergency, the medical records of this visited hospital will be photographed or scanned by the intervention clinician to preserve the data.

Participants will be requested to visit Peking University First Hospital every 3 months for 1 year (V2–V5). At each visit, exacerbations within the past 3 months, physical examinations, asthma assessment scales and lung function tests (bronchodilator reversibility tests) will be repeated. FeNO measurement will be repeated only at the final visit (V5). Exacerbation situations will include moderate exacerbations (which are defined as the use of relief therapy for more than 2 days) and severe exacerbations (which are defined as the occurrence of unplanned outpatient visits, emergency visits and hospitalizations). The exacerbation situation will be verified by medical records from Peking University First Hospital and other hospitals. All these visits (V0–V5) and data will be recorded in the case report form (CRF). Details of the follow-up visits are shown in Table 2.

**Table 2:**
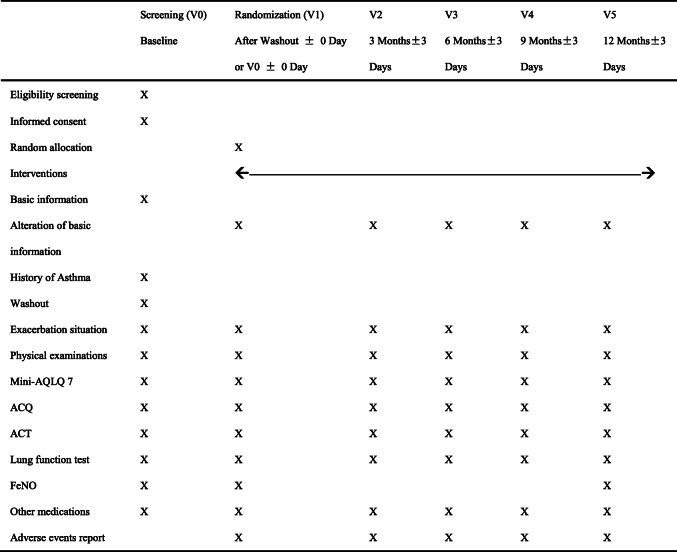
Details of the follow-up visits

*mini-AQLQ 7* Mini Asthma Quality of Life Questionnaire 7, *ACQ* Numerical Control Questionnaire, *ACT* Asthma Control Test, *FeNO* fraction of exhaled nitric oxide

The CRF is designed by the study staff. Double data entry and periodic auditing will improve data quality and integrity. Personal information and related documents of all participants will be kept strictly confidential. Every participant will be identified by a subject number and a name acronym in the CRF.

### Clinicians and blinding method

All researchers taking part in this clinical study will receive systemic training before patient enrolment. Throughout the study, the researchers responsible for the interventions and for randomization and grouping will be separated from the other researchers. As a result, the others (who will be responsible for providing medical service and measuring asthma assessment scales, for example) will be blinded to the study grouping. Data analysts will also be blinded. The data will be labelled ‘group A’ or ‘group B’ when data analysis is performed.

### Outcomes

The primary outcome is the frequency of asthma exacerbations per year, which is defined as the mean number of asthma exacerbations per patient per year at the end of the 1-year follow-up period. Asthma exacerbation situations include moderate exacerbations (defined as the use of relief therapy for more than 2 days) and severe exacerbations (defined as the occurrence of unplanned outpatient visits, emergency visits and hospitalizations).

The secondary outcomes include the mean number of unplanned outpatient visits, emergency visits, hospitalizations, medical costs and mortality caused by asthma exacerbations per patient per year at the end of the 1-year follow-up period.

### Safety and adverse events

Adverse events are unforeseeable and unfortunate events that occur during a study, either occurring with or without the intervention. Although the medicine used in our study is within the recommended dosage, adverse events may still occur during daily use. Nonsystematically via spontaneous self-report will be used to collect adverse events. All adverse events will be carefully monitored, managed and tracked in a timely manner until they are properly resolved, stabilized or returned to normal. The occurrence of adverse events will be recorded from the beginning to the end of the study. There will be supervisors who come from the administrators of Peking University First Hospital. All adverse events will be reported to these supervisors. If there are any severe adverse events, they will be reported immediately to the Peking University First Hospital Institutional Review Board (IRB). Severe adverse events will be analysed every 3 months during the study by supervisors and the IRB. If there is a definite benefit (*P* < 0.01) or an obvious disadvantage (*P* ≤ 0.05), the study will be stopped after the discussion of the centre and the approval of the ethics committee.

### Sample size

According to a previous study, every increase in PM_2.5_ of 10 μg/m^3^ increased asthma-related outpatient visits by 0.65% and emergency visits by 0.49% in Beijing [8]. Every increase in PM_10_ of 10 μg/m^3^ increased the incidence of asthma exacerbations by 3–6% [9]. The Beijing Environmental Statement published in 2016 by the Beijing Environmental Protection Agency showed that the monthly mean concentration grew from approximately 70 to 150 μg/m^3^ of PM_10_ and 52 to 150 μg/m^3^ of PM_2.5_ [10]. This means that the risk of asthma exacerbations increases by at least 30% as air pollution changes. Assuming an exacerbation frequency rate ratio (RR) of 0.85 in the RIS group compared to the control group, a total of 60 subjects (30 in each group) are required to detect a 75% reduction in air pollution-related exacerbation at 90% power with a two-sided significance level of 0.05. We recruited a total of 72 subjects considering a dropout rate of 20%.

### Statistical analysis

Statistical analysis will be carried out using SPSS 14.0 software (International Business Machines Corp., New York, USA). All statistical analyses will be performed by the two-sided test. *P* values < 0.05 will be considered statistically significant (unless otherwise specified). The Poisson regression model will be used to calculate the 95% confidence interval and the RR of exacerbation frequency. Numeric variables will be presented as the mean (standard deviation) or median (minimum, maximum; or interquartile range), and categorical variables will be presented as the number of cases (percentage). The data will be analysed by the independent sample *t* test, the Wilcoxon rank sum test, the chi-square test, the continuity corrected chi-square test or Fisher’s exact test. The characteristics of the baseline will be summarized by the equilibrium test. Unplanned outpatient visits, emergency visits, hospitalizations, medical costs and mortality caused by asthma exacerbations per year will be compared between the two groups. We have no imputation plans for missing data. The population of intention-to-treat analysis is defined as participants who have completed randomization. The population of per-protocol analysis is defined as participants who have strictly observed the intervention protocol and completed the follow-up. The population of safe set analysis is defined as participants who have completed randomization. We have no plans to conduct a modified intention-to-treat analysis.

## Discussion

This study proposes a rescue intervention strategy for asthma patients under severe air pollution. This single-centre, prospective, randomized and standard treatment parallel control clinical trial aimed to determine whether the rescue intervention strategy will reduce the risk of air pollution-related asthma exacerbations.

ICS and LABA are highly recommended by GINA for asthma patients [11]. ICS is regarded as the most important and effective drug for asthma control, although a high dose of ICS may increase the risk of pneumonia. Compared with salmeterol/fluticasone, a recent study showed that budesonide/formoterol had a lower risk of adverse events [12]. Jenkins et al. demonstrated that high-dose budesonide/formoterol (1280 μg/36 μg/day) was effective and well tolerated in asthma patients [13]. In our study, the rescue intervention strategy used is budesonide/formoterol (160 μg/4.5 μg/dose, 1 dose/time, b.i.d.) plus the original treatment until severe pollution ends (AQI < 200). The maximal dosage of budesonide/formoterol per day in our study is less than the dosage used in Jenkins’ research. Budesonide/formoterol shows rapid-acting effects in asthma and can be used in single inhalers for maintenance and relief therapy [14]. The SMART study reported that receiving budesonide/formoterol might significantly reduce the risk of asthma exacerbations [4]. As a result, budesonide/formoterol is believed to be an ideal rescue intervention drug and might be safe as an addition to the original treatment. To decrease the effects of different inhaled drugs, the original treatment for the participants in our study will be selected from the following: no use of inhaled medication, the use of SABA on demand or the use of budesonide/formoterol (160 μg/4.5 μg/dose, 1–2 dose/time, b.i.d.).

In our study, the rescue intervention strategy is to be administered until the end of severe pollution (AQI < 200). However, an AQI of 151–200 is moderate air pollution, and an AQI of 101–150 is slight air pollution. In 2018, the number of days with an AQI ≥ 100 was 138 days (37.8%), and the number of days with an AQI ≥ 200 was 15 days (4.1%) in Beijing [15]. If we take the AQI as < 100, there will be more than 100 intervention days. A recent study suggested a possible association between respiratory tract infection and the use of ICS in asthma patients [16]. As a result, the strategy we used will decrease the dosage of ICS to reduce the infection risk.

The article by Zhou et al. 2019 [6] is a protocol for a chronic obstructive pulmonary disease (COPD)-associated study that is being led by our department (Department of Respiratory and Critical Care Medicine, Peking University First Hospital). These two studies share some of the same research members, such as Guangfa Wang and Tianyu Zhou. However, the assessment scales, therapeutic replacement schemes for the washout period, inclusion and exclusion criteria, randomization, grouping etc. are suitable for asthma and single-centre studies but not for COPD and multicentre studies. Moreover, there are several differences between these two studies in terms of the intervention used. First, our study uses real-time AQI instead of 24-h mean AQI, and the rescue intervention strategy of our study will be performed until the end of severe pollution (AQI < 200) instead of the third day after the end. The HEART study found that air pollution led to a delayed inflammatory burst in the lung that lasted almost 3 days, and airway inflammation in COPD patients worsened after exposure to severe air pollution [17–19]. Nevertheless, our study uses real-time AQI, and the intervention programme may be started within the first few hours of when severe air pollution begins. This strategy might stop the inflammatory responses in an early stage to protect airways against damage from atmospheric pollutants. Moreover, a possible association between respiratory tract infection and the use of ICS in asthma patients has been reported [16]. Our strategy will decrease the dosage of ICS to reduce the risk of respiratory infection. Second, communication between the participants and intervention researchers will mainly occur through WeChat. WeChat is the most popular social app in China [20]. The use of WeChat is both customary (to improve the compliance of participants) and objective (to ensure the authenticity of the data).

The limitations of our study are as follows. First, the study does not follow a double-blind design because participants in the control group will not be administered a placebo. To decrease the potential bias, there are independent groups of researchers responsible for the intervention and for randomization and grouping. The other researchers (who are responsible for providing medical service and measuring asthma assessment scales at the follow-up visits, for example) will be blinded to the grouping results. Second, the study was not a multicentre design and was performed only in Beijing. Thus, selection bias cannot be avoided.

## Conclusions

This is a single-centre, prospective, randomized and standard treatment parallel control study aimed at decreasing the risk of asthma exacerbations under severe air pollution with a novel rescue intervention strategy.

## Trial status

This document is based on version 1.2 (11 November 2018) of the study protocol. The recruitment has finished (from 1 January 2019 to 30 June 2019), and the trial is currently at the stage of participant follow-up visits and data collection (from 1 January 2019 to 30 June 2020).

## Data Availability

Data sharing is not applicable to this article, as no datasets were generated or analysed during the current study. When the trial is completed, we plan to publish the results in a peer-reviewed journal article. The data from the trial will be available by request with privacy protection (contact with Junjun Huang, jaglpc@126.com).

## Abbreviations

AQI: Air quality index
ICS: Inhaled corticosteroids
LABA: Long-acting β-agonists
RIS: Rescue intervention strategy
SABA: Short-acting β-agonists
SMART: Single maintenance and relief therapy
SPIRIT: Standard Protocol Item Recommendations for Interventional Trials
BMI: Height, weight, body mass index
mini-AQLQ 7: Mini Asthma Quality of Life Questionnaire 7
ACQ: Numerical Control Questionnaire
ACT: Asthma Control Test
FeNO: Fraction of exhaled nitric oxide
CRF: Case report form
IRB: Institutional Review Board
PM: Particulate matter
RR: Rate ratio
GCP: Good Clinical Practice
